# In Silico Prediction of O^6^-Methylguanine-DNA Methyltransferase Inhibitory Potency of Base Analogs with QSAR and Machine Learning Methods

**DOI:** 10.3390/molecules23112892

**Published:** 2018-11-06

**Authors:** Guohui Sun, Tengjiao Fan, Xiaodong Sun, Yuxing Hao, Xin Cui, Lijiao Zhao, Ting Ren, Yue Zhou, Rugang Zhong, Yongzhen Peng

**Affiliations:** 1 Beijing Key Laboratory of Environmental & Viral Oncology, College of Life Science & Bioengineering, Beijing University of Technology, Beijing 100124, China; fantengjiao2014@emails.bjut.edu.cn (T.F.); sunxd@emails.bjut.edu.cn (X.S.); haoyuxing@emails.bjut.edu.cn (Y.H.); cuixin1201@emails.bjut.edu.cn (X.C.); renting@bjut.edu.cn (T.R.); lifesci@bjut.edu.cn (R.Z.); 2 State Key Laboratory of Bioactive Substances and Functions of Natural Medicines, Institute of Materia Medica, Chinese Academy of Medical Sciences & Peking Union Medical College, 2A Nanwei Road, Beijing 100050, China; zhouyue@imm.ac.cn; 3 National Engineering Laboratory for Advanced Municipal Wastewater Treatment & Reuse Technology, Engineering Research Center of Beijing, Beijing University of Technology, Beijing 100124, China; pyz@bjut.edu.cn

**Keywords:** MGMT, anticancer alkylating agents, resistance, inhibitors, QSAR, classification

## Abstract

O^6^-methylguanine-DNA methyltransferase (MGMT), a unique DNA repair enzyme, can confer resistance to DNA anticancer alkylating agents that modify the O^6^-position of guanine. Thus, inhibition of MGMT activity in tumors has a great interest for cancer researchers because it can significantly improve the anticancer efficacy of such alkylating agents. In this study, we performed a quantitative structure activity relationship (QSAR) and classification study based on a total of 134 base analogs related to their ED_50_ values (50% inhibitory concentration) against MGMT. Molecular information of all compounds were described by quantum chemical descriptors and Dragon descriptors. Genetic algorithm (GA) and multiple linear regression (MLR) analysis were combined to develop QSAR models. Classification models were generated by seven machine-learning methods based on six types of molecular fingerprints. Performances of all developed models were assessed by internal and external validation techniques. The best QSAR model was obtained with Q^2^_Loo_ = 0.83, R^2^ = 0.87, Q^2^_ext_ = 0.67, and R^2^_ext_ = 0.69 based on 84 compounds. The results from QSAR studies indicated topological charge indices, polarizability, ionization potential (IP), and number of primary aromatic amines are main contributors for MGMT inhibition of base analogs. For classification studies, the accuracies of 10-fold cross-validation ranged from 0.750 to 0.885 for top ten models. The range of accuracy for the external test set ranged from 0.800 to 0.880 except for PubChem-Tree model, suggesting a satisfactory predictive ability. Three models (Ext-SVM, Ext-Tree and Graph-RF) showed high and reliable predictive accuracy for both training and external test sets. In addition, several representative substructures for characterizing MGMT inhibitors were identified by information gain and substructure frequency analysis method. Our studies might be useful for further study to design and rapidly identify potential MGMT inhibitors.

## 1. Introduction

DNA alkylating agents, such as temozolomide (TMZ) and carmustine (BCNU), have been widely used for treating various malignant tumors [1,2]. These agents can undergo enzymatic hydrolysis or spontaneous decomposition to generate reactive intermediates, which act as electrophilic reagents to alkylate DNA, RNA or proteins, resulting in the loss of normal physiological function of these biomacromolecules [3,4,5,6]. Generally, they exert their anticancer activity through producing lesions at O^6^-position of DNA guanine. If not repaired correctly, these lesions can further lead to single/double-strand breaks and intrastrand/interstrand crosslinks, which inhibit strand separation during DNA replication and transcription, and ultimately result in cell apoptosis [3,5,6,7]. For example, during DNA replication, the O^6^-methylguanine (O^6^-MG) produced by TMZ mismatches with thymine forming O^6^-MG:T. Processing of O^6^-MG:T by mismatch repair (MMR) is abnormal because it recognizes the newly synthesized strand containing the thymine, leaving the O^6^-MG behind [5]. Due to the mispairing property of O^6^-MG, futile repair cycle by MMR induces DNA double-strand breaks and then leads to cell death [5,8,9]. O^6^-chloroethylguanine initially produced by chloroethylating agents subsequently rearranges to form N1,O^6^-ethanoguanine intermediate, which further reacts with the complementary cytosine to produce a G-C interstrand crosslink within several hours [3,10,11,12]. These G-C crosslinks are very poorly repaired and thus are highly toxic in mammalian cells.

However, a unique DNA repair enzyme, O^6^-methylguanine-DNA methyltransferase (MGMT), can remove the alkyl groups from the O^6^-position of guanine to the active center Cys145 residue of the protein. After accepting the alkyl groups, MGMT is inactivated and rapidly degraded by ubiquitin proteolytic pathway due to a conformational change [2,13]. In normal tissues, MGMT expression protects cells from the mutagenic or cytotoxic effects produced by environmental carcinogens or chemotherapeutic agents, whereas in tumor tissues, MGMT-mediated repair promotes resistance thereby reducing the effects of chemotherapies that alkylate the O^6^-position of guanine [3,13,14]. Previous studies have demonstrated that an inverse relationship existed between the MGMT contents and survival of malignant tumor patients treated with O^6^-guanine alkylating agents [15,16,17]. Therefore, MGMT is considered as an attractive target for cancer chemotherapy. Over the past few decades, a range of MGMT inhibitors were synthesized and used for improving the chemotherapeutic effects of these alkylating agents [2,9,13]. Unfortunately, only two compound, O^6^-benzylguanine (O^6^-BG) and O^6^-(4-bromothenyl)guanine (O^6^-4-BTG) have entered clinical trials so far [2,9]. This situation increases the importance for seeking more novel potent compounds as MGMT inhibitors.

Quantitative structure activity relationship (QSAR) and classification methods can describe a mathematic relationship between structural attributes or features and a property of chemicals [18]. In pharmaceutical industry, QSAR and classification models can be used for rapidly screening potent drug candidates from chemical databases before their synthesis, which can reduce unnecessary chemical synthesis, biological activity tests and animal experiments [18]. This appears attractive to chemical and drug manufacturers, and government agencies, especially in times of shrinking resources.

In this study, a total of 134 base analogs were utilized to establish QSAR and classification models based on their ED_50_ values (50% inhibitory concentration) against MGMT, respectively. Quantum chemical descriptors and Dragon descriptors were selected to describe molecular information and QSAR models were developed by genetic algorithm (GA) combined with multiple linear regression (MLR) analysis. Classification models were built using six types of molecular fingerprints with seven machine learning methods, which can classify MGMT inhibitors and non-inhibitors. Some privileged substructures responsible for MGMT inhibition were obtained with information gain and substructure analysis methods. In the context of design or discovery of novel compounds with desired MGMT inhibitory activity, not only these models offer a meaningful mechanistic interpretation, but also provide some crucial information between trends in structural modifications and respective changes of biological activity.

## 2. Results and Discussions

Due to DNA repair enzyme MGMT can repair the O^6^-lesions of guanine induced by chemotherapeutic agents that modify the guanine O^6^-position, MGMT inhibition in tumor cells is thus important for successful chemotherapy. To find more potent MGMT inhibitors, QSAR models and classification models were established to: (1) perform the quantitative and semi-quantitative predictions of MGMT inhibitory potency of base analogs, respectively; (2) gain some important descriptors or substructure information that can be used for discovering novel compounds with desirable activities.

### 2.1. QSAR Models

#### 2.1.1. Model Validation

**Figure 1:**
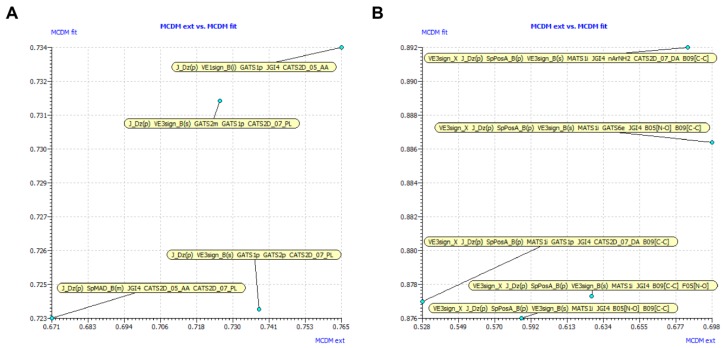
Multi-Criteria Decision Making (MCDM) graphs of the generated models based on 103 (**A**) and 84 (**B**) base analogs.

After removing constant or near-constant values and the highly inter-correlated descriptors, a further descriptor selection procedure was performed by GA combined with MLR analysis. The detailed descriptions of correlated variables were shown in Table S1 and Figure S1 in the Supplementary Materials. Then 100 possible QSAR models were produced with different predictive abilities. Models with multicollinearity were eliminated after utilizing QUIK (Q Under Influence of K) module [19]. For acceptable QSAR predictive models, the following conditions are satisfied [20,21]: (i) Q^2^_Loo_ > 0.5; (ii) R^2^_ext_ > 0.6; (iii) (R^2^_ext_ − R_0_^2^)/R^2^_ext_ < 0.1 and 0.85 ≤ *k* ≤ 1.15 or (R^2^_ext_ − R’_0_^2^)/R^2^_ext_ < 0.1 and 0.85 ≤ *k’* ≤ 1.15; (iv)|R_0_^2^ − R’_0_^2^| < 0.3. R_0_^2^ and R’_0_^2^ represent correlation coefficients of experimental versus predicted values and predicted versus experimental values for regressions through the origin, respectively. *K* and *k’* are the corresponding slopes of regression lines through the origin. Finally, Multi-Criteria Decision Making (MCDM) was used to rank the performance of models as scores and select candidate models (Figure 1) [22].

For initial QSAR modeling based on 103 base analogs, four models were chosen, which Q^2^_Loo_ and R^2^ values ranged from 0.6293 to 0.6367 and 0.6701 to 0.6814, respectively, as listed in Table S2 in the Supplementary Materials. The best QSAR model (**I**) with five model descriptors for predicting MGMT inhibitory activity was obtained as below:

pED50 (−LogED_50_) = 12.80 − 4.39J_Dz(p) − 8.05VE1sign_B(i) − 5.02GATS1p + 104.16 JGI4 − 0.39CATS2D_05_AA (1)

N_tr_ = 83, Q^2^_Loo_ = 0.64, R^2^= 0.68, R^2^_adj_ = 0.66, F = 32.94, RMSE_tr_ = 0.87, CCC_tr_ = 0.81;

N_test_ = 20, Q^2^_ext_ = 0.76, R^2^_ext_ = 0.78, RMSE_test_ = 0.75, Q^2^_F1_ = 0.76, Q^2^_F2_ = 0.76, Q^2^_F3_ = 0.77, CCC_test_ = 0.88, (R^2^_ext_ − R_0_^2^)/R^2^_ext_ = 0.01, *k* = 0.97, (R^2^_ext_ − R’_0_^2^)/R^2^_ext_ = 0.02, *k’* = 1.01, |R_0_^2^ − R’_0_^2^| = 0.007.

For further QSAR modeling based on 84 base analogs, five models were chosen, which Q^2^_Loo_ and R^2^ values ranged from 0.8000 to 0.8266 and 0.8385 to 0.8724, as listed in Table S3 in the Supplementary Materials. The best QSAR model (II) with nine model descriptors for predicting MGMT inhibitory activity was obtained as below:

pED50 (−LogED_50_) = 19.47 − 0.17VE3sign_X − 6.04J_Dz(p) + 22.5SpPosA_B(p) + 0.28VE3sign_B(s) + 3.05MATS1i + 138.84JGI4 + 0.60nArNH2 − 0.19CATS2D_07_DA − 1.36B09[C-C] (2)

**Figure 2:**
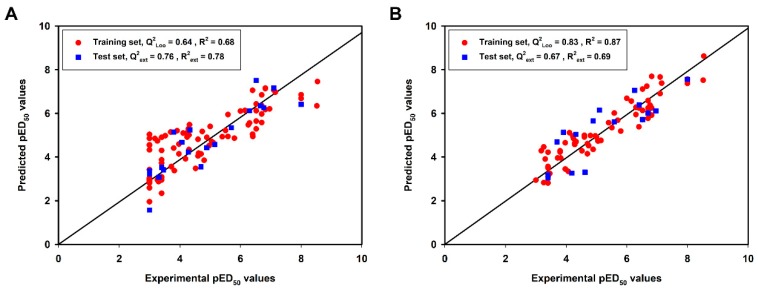
Plots of the experimental versus predicted pED_50_ values for compounds in the training set and test set of the best models derived from initial (**A**) and further (**B**) quantitative structure activity relationship (QSAR) modeling.

N_tr_ = 68, Q^2^_Loo_ = 0.87, R^2^= 0.87, R^2^_adj_ = 0.85, F = 44.06, RMSE_tr_ = 0.53, CCC_tr_ = 0.93;

N_test_ = 16, Q^2^_ext_ = 0.67, R^2^_ext_ = 0.69, RMSE_test_ = 0.79, Q^2^_F1_ = 0.67, Q^2^_F2_ = 0.67, Q^2^_F3_ = 0.71, CCC_test_ = 0.83, (R^2^_ext_ − R_0_^2^)/R^2^_ext_ = 0.04, *k* = 0.9885, (R^2^_ext_ − R’_0_^2^)/R^2^_ext_ = 0.03, *k’* = 0.99, |R_0_^2^ − R’_0_^2^| = 0.005;

N_tr_ and n_test_ mean the number of compounds in the training set and test set, respectively. For both models, the values of Q^2^_Loo_, R^2^, R^2^_adj_ and RMSE for the training sets indicated the good internal fitting ability and robustness. The test set compounds, which were not included in modeling, were used for an external validation to confirm the predictive ability of the models. The statistical parameters for the test sets (Q^2^_ext_, R^2^_ext_, RMSE_test_) showed good external predictive performances of two models. The high Q^2^_F1_, Q^2^_F2_, Q^2^_F3_ and CCC_ext_ values also indicated these two models had good external prediction. Furthermore, significantly lower statistical values of R^2^_Yscr_ and Q^2^_Yscr_ were observed in Y-scrambling procedure when compared to the original models (Tables S2 and S3 in the Supplementary Materials), thus we considered that the proposed QSAR models were not obtained by chance. A good accordance between predicted and experimental values was reflected by the homogenous distribution around the optimal line (Figure 2).

#### 2.1.2. Outliers Analysis and Applicability Domain of QSAR Models

**Figure 3:**
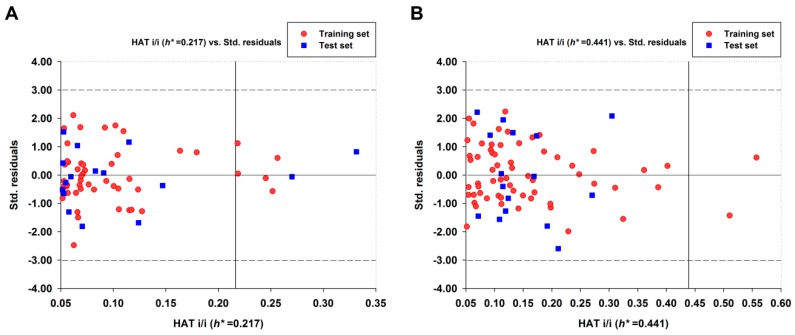
Williams plots for the best QSAR models based on model **I** (**A**) and model **II** (**B**). The transverse dash lines represent ±3 standard residual and vertical black line represents warning leverage h*.

QSAR models are only valid and functional with a given Applicability Domain (AD). The AD of the best QSAR models were presented by Williams plots (Figure 3). As shown in Figure 3, no response outliers were observed for both two QSAR models because the predicted activities of all compounds were lower than ±3 standardized residuals, suggesting that the MGMT inhibitory activity of base analogs were reliably predicted by models I and II. For model I, it is important to note that seven compounds (5 in training set and 2 in test set) exhibited higher leverage values (*h*) than critical hat value (h* = 0.217) (Figure 3A). By contrast, only two compounds in training set were observed with higher *h* values than h* value (0.441) for model II and all test set compounds fell in the structural AD of model II (Figure 3B). For those compounds having high *h* values in the data set, predictions could be unreliable, although prediction performance is also good (no outlier was found). These results suggested that model II based on 84 base analogs had better predictive power for MGMT inhibition than that of model I based on 103 base analogs. Therefore, model II was further analyzed in the next mechanism interpretation.

#### 2.1.3. Mechanism Interpretation

Equation (2) indicated that model II included the following nine molecular descriptors: VE3sign_X, J_Dz(p), SpPosA_B(p), VE3sign_B(s), MATS1i, JGI4, nArNH2, CATS2D_07_DA, and B09[C-C]. Molecular descriptors included in model II, corresponding types and chemical meanings were given in Table 1, and the detailed explanation can be found in Handbook of Molecular Descriptors [23]. Regarding the coefficients in Equation (2), five descriptors (SpPosA_B(p), VE3sign_B(s), MATS1i, JGI4 and nArNH2,) exhibited positive contribution and four descriptors (VE3sign_X, J_Dz(p), CATS2D_07_DA and B09[C-C]) had negative contribution to MGMT inhibition. JGI4 is mean topological charge index of order 4. Topological charge indices were proposed to measure the charge transfer between pairs of atoms, and consequently, the global charge transfer in the molecule (e.g., the dipole moment) [24,25]. Compounds with increased MGMT inhibitory activities were tended to have high dipole moment (JGI4 values). The importance of charge transfer has already been reported in other study [26]. SpPosA_B(p) means normalized spectral positive sum from Burden matrix weighted by polarizability, is another important variable positively correlates with the increased MGMT inhibition of base analogs. Polarizable molecules are commonly regarded as ‘soft’ species that are prone to attack other soft species, therefore, it seems that more-polarizable molecules are prone to have higher activities/toxicities. This might be caused by the formation of covalent bonds involving the soft acids and bases [27]. Most active compounds against MGMT were observed with high polarizability. In addition, VE3sign_B(s), MATS1i and nArNH2 characterizing I-State, ionization potential (IP) and number of primary aromatic amines, respectively, also gave positive contributions for MGMT inhibitory activities of base analogs. For example, nArNH2 implies the number of primary aromatic amines, its positive effect for MGMT inhibition may be due to the promotion of hydrogen bond formation. This assumption has been proposed in previous studies, where O^6^-(3-aminomethyl)benzylguanine (**57**) exhibited more potency than O^6^-BG in inhibiting MGMT, because the aminomethyl group in **57** can form another hydrogen bond with MGMT compared to O^6^-BG [28,29]. MATS1i represents the Moran autocorrelation of lag 1 weighted by IP, which is a measure of the ability of a molecule to give the corresponding positive ion. In fact, MGMT inhibition by base analogs is mediated by the removal of carbonium ion to its active center Cys145 residue of the protein [3,13]. It has been demonstrated that O^6^-BG having a benzyl group was observed with higher MGMT inhibitory potency than O^6^-MG possessing a methyl group, this is due to benzyl group is more easily displaced in bimolecular displacement reactions than methyl group [13]. MATS1i descriptor has also been used in the prediction of acute toxicity of alkylbenzenes and antitumor activity of benzensulfonamide derivatives [30,31], where the biological responses or activities increased with the high values of MATS1i descriptor, which are consistent with our finding. J_Dz(p) represents balaban-like index from Barysz matrix weighted by polarizability, the negative coefficient indicates it is inversely related to MGMT inhibition. A similar result was obtained between the anti-protozoal activity of novel polyamine analogs and J_Dz(p) [32]. VE3sign_X, determining logarithmic coefficient sum of the last eigenvector from chi matrix, the negative coefficient indicates the increase in the value of this descriptor may result in a slight decrease in MGMT inhibitory potency. CATS2D_07_DA is a 2D structure-based atom-pair descriptor which defines potential pharmacophore points of hydrogen bond donor/acceptor at the topological distance of 7 (i.e., a distance of seven bonds). The coefficient of CATS2D_07_DA shows this descriptor negatively correlates with −logED_50_. The last descriptor B09[C-C], a 2D binary fingerprint, representing the presence/absence of C-C at topological distance 9. The negative coefficient of this descriptor implies that the value of B09[C-C] is inversely proportional to the MGMT inhibition, this is confirmed that all most of the active compounds have low values of B09[C-C].

### 2.2. Classification Models

#### 2.2.1. Data Set Analysis

After original data screening, a total of 129 base analogs collected from available literature were randomly divided into a training set for building models and an external test set for validating the quality of the proposed models in the ratio of 4:1. According to the classification criterion described in Materials and Methods, a dataset with 62 positive and 67 negative MGMT inhibitors was obtained, in which the training set contained 50 inhibitors and 54 non-inhibitors while the test set contained 12 inhibitors and 13 non-inhibitors (Table S4 in the Supplementary Materials). It should be noted that each set contained almost the same proportion of potential MGMT inhibitors (training set = 48.1%, test set = 48%).

**Figure 4:**
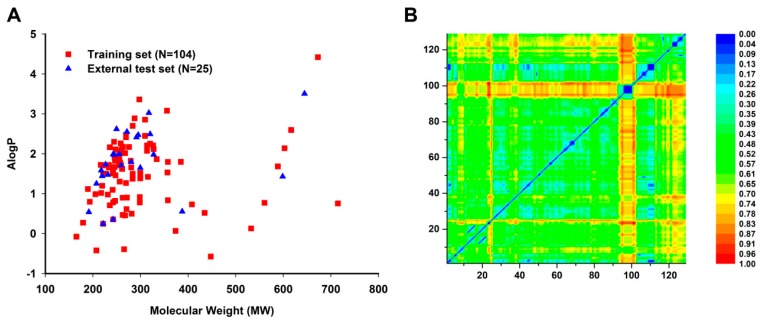
Chemical diversity distribution of the training set (N = 104 compounds), external test set (N = 25 compounds). (**A**) Chemical space was analyzed using the molecule weight (MW) and Ghose-Crippen LogKow (ALogP) of each set in the database. N represents the number of compounds in each data set. (**B**) Heat map of molecular similarity plotted by Euclidian distance metrics for the training and external test sets. Euclidian distance metrics was calculated by MACCS keys fingerprint and processed by normalization.

To develop a robust and reliable prediction model, we performed the chemical diversity analysis of this novel data set. The chemical space distribution was analyzed using the molecule weight (MW) and Ghose-Crippen LogKow (ALogP) of each set in the database [33,34]. The scatter plot constructed by MW and ALogP was illustrated in Figure 4A. As shown in Figure 4A, all external test set compounds possessed similar chemical space with the training set. Euclidian distance metrics of the whole data set was calculated using MACCS keys fingerprint to further evaluate the chemical diversity of compounds in the two sets. The heat map of the Euclidian distance metrics was presented in Figure 4B, where the training set and the test set were compared with each other. It was obvious that the data set was chemically diverse.

#### 2.2.2. Performances of 10-Fold Cross-Validation

**Figure 5:**
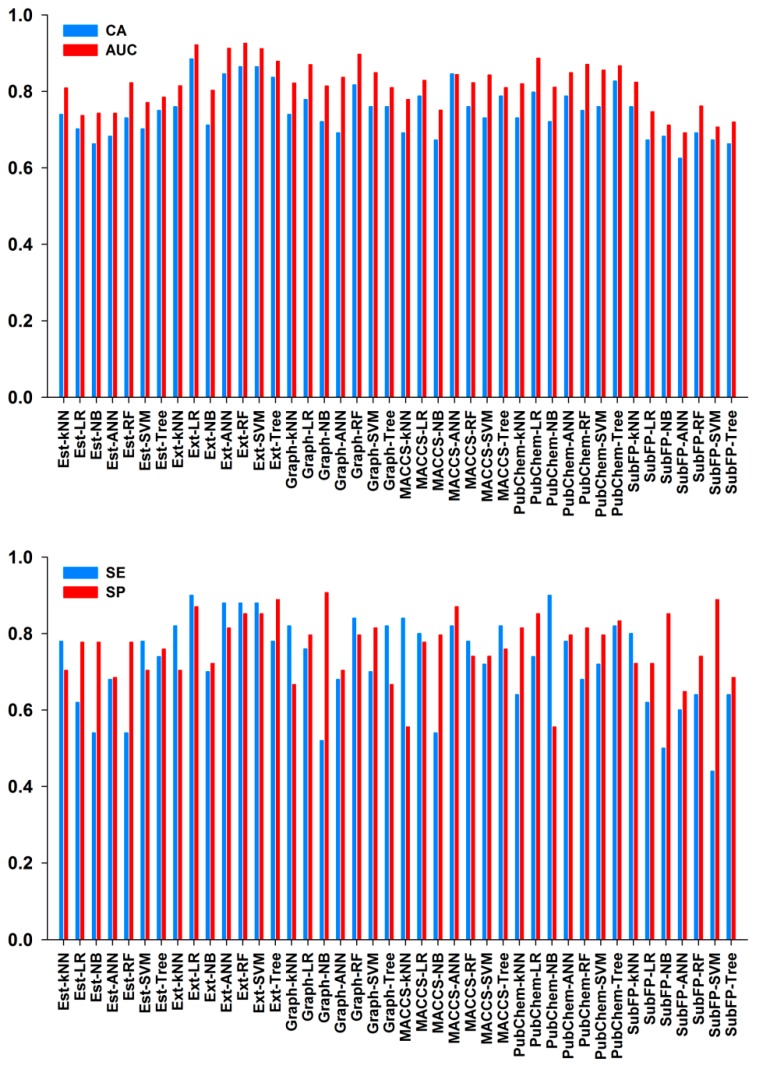
Performance of 10-fold cross-validation for the training set in 42 classification models. CA, AUC, SE and SP represent the classification accuracy; the area under the ROC curve, sensitivity and specificity, respectively.

In classification study, the binary classification models were established using six molecular fingerprints combined with seven machine learning methods, including *k*-nearest neighbor (*k*NN), Logistic Regression (LR), Naïve Bayes (NB), artificial neural network (ANN), support vector machine (SVM), Random Forest (RF) and Tree. Eventually, a total of 42 predictive models were generated based on the training set. 10-Fold cross-validation was performed to evaluate the performance of all models, and the best models were chosen according to the values of classification accuracy (CA) and the area under the ROC curve (AUC). Figure 5 presented the detailed evaluation results of these classification models. As can be seen in Figure 5, the CA and AUC values in all models were observed with more than 0.6, where CA values ranged from 0.625 to 0.885 and AUC values ranged from 0.692 to 0.926. Overall, the Ext fingerprint exhibited the best performance, while the SubFP fingerprint gave the worst effect of classification when the same algorithm was used. Ext molecular fingerprint is an extension of the Chemistry Development Kit (CDK) fingerprint with bits that take into account ring features, and the length of Ext fingerprint is 1024, which is full of structural information [35]. Ext fingerprint is well fit for predicting and gaining insight into drug activity. For example, Ext fingerprint has been proven to perform well for classifying predicting androgen or estrogen receptors binders or non-binders and acetylcholinesterase inhibitors or noninhibitors, respectively [36,37]. Based on the results, the top ten models were Ext-RF, Ext-LR, Ext-ANN, Ext-SVM, Graph-RF, PubChem-LR, Ext-Tree, PubChem-RF, Graph-LR and PubChem-Tree, respectively. For the top ten models, their CA values were 0.750−0.885 and AUC values were 0.867−0.926. Except for PubChem-RF model, most models had SE values higher than 0.7. It was inspiring that all top ten models were observed with SP values higher than 0.8. No significant difference was found between SE and SP values, suggesting that these models had good predictive ability for both inhibitors (P) and non-inhibitors (N). Table 2 listed the detailed performance of the top ten models for training and test sets. According to the results of the 10-fold cross-validation, we could obtain three conclusions. The first one was that most models exhibited good overall predictive performance for the training set. The second one was that different molecular fingerprints greatly differed in prediction ability when the same machine learning method was performed. The third one was that the models with good performance were mainly constructed using the Ext fingerprint combined with different algorithms. Among these models, Ext-RF model produced the best result (CA = 0.865, AUC = 0.926, SE = 0.88, SP = 0.85).

#### 2.2.3. Performances of External Test Set

The top ten models were further validated by the external test set. Table 2 also listed the detailed results of the ten best models for external test set. Their CA values were 0.667−0.875 and AUC values were 0.626−0.992. Except for PubChem-Tree model (CA = 0.640, AUC = 0.667), all models showed good external predictive performance with CA values higher than 0.8 and AUC values higher than 0.9. Similar to the training set, there were no significant differences between the SP (0.62–0.92) and SE (0.67–0.92) values for test set in these models, which reflected nearly identical predictive ability for “P” and “N” inhibitors in these models. Among these models, Ext-SVM, Ext-Tree and Graph-RF models provided the best results with the highest overall accuracy of 88%, other six models (Ext-RF, Ext-LR, Ext-ANN, PubChem-LR, PubChem-RF and Graph-LR) also gave high predictive accuracy (≥80%). Meanwhile, they also shared good predictive performance for each class (“P” and “N”). We proposed that the good predictive ability for each class might be due to the balanced distribution of inhibitors and non-inhibitors with a ratio of 0.92. Considering the SE and SP values, among the nine candidate models, Ext-ANN model (SE = 0.67, SP =0.92) was not good enough due to the imbalance of predictive power for “P” and “N” inhibitors. Overall, the prediction results for external test set revealed the stable robustness and precise prediction accuracy of the models. In view of the prediction accuracy, Ext fingerprint was recommended for developing the in silico predictive models for MGMT inhibition. 

#### 2.2.4. Identification and Analysis of Privileged Substructures

To investigate structural features of base analogs as inhibitors and non-inhibitors of MGMT, information gain (IG) and substructure frequency analysis methods were used for identifying privileged substructures in both the training and external test sets using the PubChem fingerprint [38]. Detailed results of IG values and frequencies of each fragment occurred in the “P” and “N” classes were listed in Table S5 in the Supplementary Materials. Some representative substructures and compounds containing these substructures were presented in Table 3. As shown in Table 3, we found 21 privileged substructures, which correspond to nine general substructures (2-bromoprop-1-ene, 2-bromobuta-1,3-diene, thiophene, *p*-tolylmethanol, ≥2 saturated or aromatic heteroatom-containing ring size 6, *E*-2-nitroethenamine, ≥3 hetero-aromatic rings, *p*-xylene, *m*-xylene), were more frequently appeared in MGMT inhibitors than non-inhibitors. This implies compounds containing these substructures have more potency to inhibit MGMT. In other words, these substructures can be considered as structural alerts for high MGMT inhibitory potency. For example, O^6^-benzyl-8-bromoguanine (47) containing a 2-bromoprop-1-ene fragment, O^6^-thenylguanine (62) containing a thiophene fragment and O^6^-(3-aminomethyl)benzylguanine (57) containing a *m*-xylene fragment were three potent MGMT inhibitors with high activities. In this study, compounds having both the Br atom and thiophene group were all potent MGMT inhibitors. The saturated six-membered ring glucosyl or aromatic heterocycle also contribute to the formation of hydrogen bond with MGMT, in which glucosyl conjugation preferentially targets tumor cells [29,39]. The positive effect of nitroethenamine fragment may be caused by the nitro group which makes the arylmethyl group more reactive to the active site Cys145 residue of MGMT [40]. It is worth noting that previous studies have proposed that the *meta*-substitution of aminomethyl in compound 57 promoted additional hydrogen bond formation with Asn137 residue of MGMT compared to standard inhibitor O^6^-BG (2), which resulted in increased inhibitory ability of compound 57 [28,29]. The favorable effects of *meta*-substitution of xylene were also reflected in compound 60, 109, 112 and 128 etc. The *p*-tolylmethanol substitution in base analogs performed well is due to the steric effect of the MGMT active pocket, which has been proposed in our previous study [29].

## 3. Materials and Methods

### 3.1. QSAR Study

#### 3.1.1. Data Set

In this study, a total of 134 base analogs with different inhibitory activity against MGMT were carefully collected from previously published literatures [28,29,39,40,41,42,43,44,45,46,47,48]. Among these compounds, 26 compounds (109~134) were not included in QSAR study due to different experimental conditions. Additionally, five compounds (72, 73, 75, 79, and 86) were also not used for establishing QSAR models since they were untested. Compounds 13 and 19 (sodium salts) were converted to corresponding carboxylic acids. Finally, a set of 103 base analogs were used as a data set for initial QSAR modeling. Considering that 19 compounds were observed with ED_50_ values > 1000 μM, in order to obtain more possibly reliable QSAR models, they were excluded in further QSAR modeling. Their activities were identified in vitro under the same experimental conditions, as measured by ED_50_ values, which is the dose required to produce 50% inactivation of MGMT. Most regression algorithms depend on the data being normally distributed, so if the data are not normally distributed, a logarithmic transformation should be applied to obtain a normal distribution. All original data were expressed as pED_50_ values (pED_50_ = −logED_50_) and were used as the dependent variables in QSAR study. The pIC_50_ values span more than 5 log units, indicating an adequate dataset for a QSAR study. All compounds were ranked according to their pED_50_ values, then one was picked out of every five compounds to constitute test set and the remaining compounds were used as training set. The chemical structures and experimental activities of the compounds were listed in Table S6 in the Supplementary Materials.

#### 3.1.2. Calculation of Molecular Descriptors

Molecular structures of all compounds were generated by Gaussview 5.0 software, and then optimized by density functional theory (DFT) method using the Gaussian 09 program with Becke’s three-parameter exchange potential and the Lee-Yang-Parr correlation functional (B3LYP) and 6-311++G(d,p) basis set [49]. Frequency analyses were performed to ensure that the optimized geometries were their corresponding local minima. After geometry optimization, a set of quantum chemical descriptors were calculated, including dipole moment (μ), total energy (E), the highest occupied molecular orbital energy (*E*_HOMO_), the lowest unoccupied molecular orbital energy (*E*_LUMO_), *E*_LUMO_ − *E*_HOMO_ gap, the bond lengths and the bond angles. 

After structure optimization, Dragon descriptors were calculated by DRAGON software (version 7.0) [50]. Due to most of 3D descriptors encoding 3D structures were found to be sensitive to the quantum chemical calculation method which may influence the quality of QSAR model, thus we removed the 3D descriptors [51]. DRAGON 7.0 contains 22 2D molecular descriptor blocks (e.g., constitutional indices, ring descriptors, topological indices, connectivity indices, and so on), which consist of a total of 3822 0-2D descriptors. The wide range of descriptors will facilitate the discovery of hidden important variables. Subsequently, we performed pre-filtration to exclude the constant or near-constant values (>80%) and the highly inter-correlated descriptors (>95%). Finally, the remaining 520 Dragon descriptors were combined with the quantum chemistry descriptors to establish the QSAR models.

#### 3.1.3. Model Development and Evaluation

QSAR models were generated by QSARINS 2.2.2 software (Varese, Italy) [22,52] with MLR method. Descriptor selection was carried out by all subsets and GA tools of QSARINS 2.2.2 software. To avoid a completely random start of the GA, all low-dimensional models (up to 2–3 descriptors) were first calculated using the all subset facility to gain an insight into the best descriptors encoding the response. The best subset of descriptors determined at this step was used as the core of chromosomes of the initial population for the GA. Then, GA was used to explore the solution space by maximizing the leave-one-out (LOO) cross-validation correlation coefficient (Q^2^_Loo_) as the fitness function. The population size, mutation rate and number of generations were set as 200, 20, and 2000, respectively. According to the rule-of-thumb [21,53], the ratio of the number of compounds in training set to the number of selected descriptors should be at least 5, which suggests that at most 16 or 13 descriptors are allowed in initial or further QSAR study. Q^2^_Loo_ is a crucial parameter to evaluate model stability and robustness. Following this procedure repeatedly, a population of good models were generated.

The goodness-of-fit and robustness of QSAR models were evaluated by the Q^2^_Loo_, correlation coefficient R^2^, modified form of R^2^_adj_, and root mean square error (RMSE). Inter-correlation of descriptors was tested via the QUIK rule [19], which was set to 0.05 to avoid models with multicollinearity. The possibility of chance correlation in the QSAR models was also checked by a Y-scrambling procedure (2000 iterations to check the fitting of the randomly reordered Y-data) [21,54]. If the new QSAR models obtained by randomly shuffling the pED_50_ values generate significantly lower Q^2^_Loo_ than the original model, we considered that the proposed QSAR model was not obtained by any chance correlation.

The compounds in the test set, which are not used in model development, were used to assess the external predictive ability of the models by Q^2^_ext_ and R^2^_ext_. Q^2^_ext_ = 1 − PRESS/SD, where PRESS means the sum of squared deviations between the experimental value and the predicted value for each compound in the test set, and SD means the sum of squared deviations between the experimental values of the test set molecules and the mean experimental value of the training set compounds [20]. Q^2^_F1_ [55], Q^2^_F2_ [56], Q^2^_F3_ [57,58], Concordance Correlation Coefficient (CCC) [59,60], CCC_ext_ [61,62] and RMSE_ext_ are also involved.

MCDM method implemented in QSARINS 2.2.2 software was utilized to rank the performances of models as scores [22]. The score is between 0 to 1, in which 0 and 1 imply the worst and the best validation criteria, respectively. After multiple rounds of trials, model was finally chosen via the best MCDM score, which should satisfy the statistical standard for fitting, internal and external validations, and with the least possible number of descriptors.

#### 3.1.4. Applicability Domain

In order to understand the scope and limitations of the proposed QSAR models, applicability domain (AD) was considered. Only compounds falling in the AD of the model, their predicted values are considered as reliable. The AD of each model was assessed with a leverage approach [54]. The leverage of a compound in the original variable space is defined as hat value (*h*). The warning leverage (*h*^∗^) is defined as *h*^∗^ = 3(p + 1)/n, where p represents the number of predictor variables and n represents the number of training compounds. For training set, compounds with *h* > *h*^∗^ seriously influence the regression parameters of models. For those compounds with *h* > *h*^∗^ in the test set, their predicted values should be unreliable. Williams plot, a plot of standardized residuals versus leverages, was used to visualize the applicability domain of a QSAR model. Response outliers were also identified if the predicted activities are higher than ±3 standardized residuals [54].

### 3.2. Classification Study

#### 3.2.1. Data Collection and Preparation

Except for five untested compounds (72, 73, 75, 79 and 86), the remaining 129 compounds were used in classification study. The salt chemicals were transformed to the corresponding acids. Based on the standard inhibitor O^6^-BG (2), compounds exhibited more than 1/50 potency of O^6^-BG were considered as MGMT inhibitors, otherwise were considered as non-inhibitors. For compounds 109~117, there were no accurate ED_50_ values when their ED_50_ values > 10 μM or < 1 μM [48]. Because the activity of O^6^-BG was identified as <1 μM under the same experimental conditions [48], so compounds with ED_50_ values >10 μM were identifies as non-inhibitors. Finally, a dataset containing 62 inhibitors and 67 non-inhibitors was obtained. MGMT inhibitors were represented as “P” and non-inhibitors as “N” when building the classification models. All compounds were then randomly divided into a training set and an external test set with a ratio of 4:1. A complete list of the compounds’ classification was presented in Table S6 in the Supplementary Materials.

#### 3.2.2. Molecular Fingerprints

Molecular fingerprints have been widely used in similarity searching and classification. Therefore, substructure features in each fingerprint dictionary are defined to contain full of representative substructures. By this method, a molecule was described as a binary string of structural keys. SMiles Arbitrary Target Specification (SMARTS) is a language capable of describing molecular patterns and properties using rules that are extensions of simplified molecular input line entry specification (SMILES) [63]. For a SMRATS pattern, if a substructure presented in the given molecule, the corresponding bit was set to “1”and otherwise set to ”0” [63]. Six fingerprints, including Extended fingerprint (Ext, 1024 bits), Estate fingerprint (Est, 79 bits), MACCS keys (166 bits), PubChem fingerprints (881 bits), CDK graph only fingerprint (Graph, 1024 bits) and Substructure fingerprint (SubFP, 307 bits) were used in our study. All these six fingerprints were calculated using the PaDEL-Descriptor software [64].

#### 3.2.3. Machine Learning Methods

Seven different methods, including *k*NN, LR, NB, ANN, SVM, RF and Tree were used for model building. All these methods were performed by Orange Canvas 3.11 software (freely available at https://orange.biolab.si/).

*k*NN. It is a nonparametric method that classifies objects depending on nearest training examples in the feature space [65]. It can be classified by a majority vote of the nearest neighbors, with the object being assigned to the class most common among its *k* nearest neighbors. In our study, Euclidean distance and distance-weighted parameters have been chosen, and the parameter of *k* = 5 was used.

LR. It was developed in 1958 by statistician David Cox [66,67]. The binary logistic model is a statistical model which is usually applied for a binary dependent variable. The dependent variable values can be labeled as symbols of “0” and “1”, which represent outcomes such as pass/fail, positive/negative or yes/no, respectively.

NB. NB has been studied extensively since the 1950s, it is a simple classification method based on the Bayes rule for the conditional probability [68,69], which allows the user to classify instances in a data set based on the equal and independent contributions of their attributes. NB generates the prior probability that is directly given out from the training set since it is the same to all of the classes, while the marginal probability is ignored. The default settings in Orange were used to perform the NB classification.

ANN. ANN has been an effective tool to identify complex nonlinear relationship between independent variables and dependent variables for classification and regression [70]. In this work, the network contained three layers, including one input layer, one hidden layer, and one output layer. ANN in Orange Canvas is a multi-layer perceptron (MLP) algorithm with backpropagation. In this study, each hidden layer included 200 neurons.

SVM. SVM was first introduced by Vapnik et al. in 1995. It is a kernel-based algorithm for binary data classification and regression [71]. Substructure pattern recognition method, which worked as an eigenvector for SVM, described each molecule as a binary string. After training, SVM could give a decision function for classification. Polynomial kernel, Gaussian radial basis function kernel (RBF) and sigmoid kernel are the commonly used functions. In this study, the RBF kernel was chosen. The parameters C and γ for RBF kernel were tuned on the training set by 10-fold cross validation. Orange embeds a popular implementation of SVM from the LIBSVM package [72]. The linear function was chosen and the cost was set to 1.00.

RF. RF, an ensemble learning method for classification and regression, was developed by Breiman [73]. The forest is assembled by trees. Each tree in the ensemble is randomly produced by first selection and a small group of input coordinates (features or variables) to split at each node. The best split is calculated based on these features from the training set. The tree is grown up to maximum size without pruning, and the forest chooses the classification depended on the majority of individual tree’s output. The number of trees in forest was set to 20.

Tree. Tree is a standard benchmark in machine learning and incorporated in Orange, which can handle both discrete and continuous datasets. It includes decision nodes, branches, and leaves. A decision tree takes as input an object or situation characterized by a number of properties and outputs a yes or no decision. An instance is classified by starting at the root node of the decision tree, testing the attribute defined by this node, and then moving down to the tree branch based on the attribute value [74]. In the pre-pruning, the minimal instance in leaves was set to 3, and stop splitting nodes with instances less than 5. Other parameters of tree in Orange were used with the default values.

#### 3.2.4. Model Performance Evaluation

All models were evaluated by 10-fold cross-validation and a diverse external test set. All the models were assessed by the counts of true positives (TP), true negatives (TN), false positives (FP), and false negatives (FN) compounds. Furthermore, sensitivity (SE), specificity (SP) and overall predictive accuracy (CA), which represent predictive accuracies of “P”, “N”, and total compounds were calculated with the following equations, respectively [34,38].

SE = TP/(TP + FN)(3)

SP = TN/(TN + FP)(4)

CA = (TP + TN)/(TP + TN + FP + FN)(5)

In addition, the receiver operating characteristic (ROC) curve where the TP rate (sensitivity) versus the FP rate (1-specificity) was plotted, and the area under the ROC curve (AUC) was also calculated to evaluate the quality of a model. The values of AUC range from 0.5 (no discriminative power) to 1.0 (perfect classifier) [75].

#### 3.2.5. Privileged Substructure Analysis

The privileged substructures were analyzed using the information gain (IG) method along with substructure fragment analysis [38,63]. If a substructure was more frequently existed in the class of “P”, this substructure was considered as a privileged substructure involved in MGMT inhibition. The IG values were calculated to evaluate and generate the final privileged substructures. The frequency of a fragment in MGMT inhibitors was defined as follows [76,77]:

(6) $$\mathrm{Frequency}\;\mathrm{of}\;a\;\mathrm{substructure}\;=\;\frac{N_{fragment}^{I}\;\times N_{total}}{N_{fragment\_total}\;\times \;N_{I}}$$

where *N^I^_fragment_* the number of compounds containing the fragment in MGMT inhibitors; *N_total_* is the total number of compounds in the data set; *N_fragment_total_* is the total number of compounds containing the fragment; and *N*_I_ is the total number of “P” in the data sets.

## 4. Conclusions

In this work, a total of 134 base analogs were used as a data set for QSAR and classification study for in silico prediction of MGMT inhibitory potency. After data processing, two QSAR models (I and II) were developed using GA-MLR methods based on 103 and 84 base analogs, respectively. The statistical parameters showed that these two models had good internal fitting and good external prediction ability. However, outliers and AD analyses of the models indicated that model II was better than that of model I. The mechanism of model II was then interpreted and the main molecular descriptors governing –logED_50_ value are the molecular ability of topological charge indices, polarizability, IP and number of primary aromatic amines in a molecule. All the classification models were established by seven machine learning methods (*k*NN, LR, NB, ANN, SVM, RF, and Tree), along with six molecular fingerprints (Ext, Est, MACCS, PubChem, Graph and SubFP). The performances of these models were evaluated by 10-fold cross-validation and an external test set containing 25 diverse compounds. Three best models for predicting the classification of MGMT inhibitors were Ext-SVM, Ext-Tree and Graph-RF that had the highest overall accuracy of 88%, and their AUC values were both higher than 0.9. IG and substructure frequency analysis were utilized to identify privileged substructures or fragments as structural alerts for potent MGMT inhibitors. As a result, nine general substructures, including 2-bromoprop-1-ene, 2-bromobuta-1,3-diene, thiophene, *p*-tolylmethanol, ≥2 saturated or aromatic heteroatom-containing ring size 6, *E*-2-nitroethenamine, ≥3 hetero-aromatic rings, *p*-xylene, *m*-xylene, were main contributors to MGMT inhibition in base analogs. Compared to QSAR models, semi-quantitative classification models could directly provide rapid identification of potent MGMT inhibitors. In conclusion, our study not only provides useful tools for in silico prediction of MGMT inhibitory potency of base analogs quantitatively or semi-quantitatively, but also is helpful to further inhibitor design targeting MGMT.

## Figures and Tables

**Table 1:** Types and chemical meanings of molecular descriptors used in model **II**.

Descriptor	Type	Chemical Meaning
**VE3sign_X**	2D matrix-based descriptors	logarithmic coefficient sum of the last eigenvector from chi matrix
**J_Dz(p)**	2D matrix-based descriptors	balaban-like index from Barysz matrix weighted by polarizability
**SpPosA_B(p)**	2D matrix-based descriptors	normalized spectral positive sum from Burden matrix weighted by polarizability
**VE3sign_B(s)**	2D matrix-based descriptors	logarithmic coefficient sum of the last eigenvector from Burden matrix weighted by I-State
**MATS1i**	2D autocorrelations	Moran autocorrelation of lag 1 weighted by ionization potential
**JGI4**	2D autocorrelations	mean topological charge index of order 4
**B09[C-C]**	2D Atom Pairs Binary	presence/absence of C-C at topological distance 9
**nArNH2**	Functional group counts	number of primary amines (aromatic)
**CATS2D_07_DA**	CATS 2D	CATS2D Donor-Acceptor at lag 07

**Table 2:** Performance of top ten binary classification models for the training and external test sets ^1^.

Data Set	Model	CA	AUC	SE	SP	TP	TN	FP	FN
Training set	Ext-RF	0.865	0.926	0.88	0.85	44	46	8	6
	Ext-LR	0.885	0.922	0.90	0.87	45	47	7	5
	Ext-ANN	0.846	0.913	0.88	0.81	44	44	10	6
	Ext-SVM	0.865	0.912	0.88	0.85	44	46	8	6
	Graph-RF	0.817	0.897	0.84	0.80	42	43	11	8
	PubChem-LR	0.798	0.887	0.74	0.85	37	46	8	13
	Ext-Tree	0.837	0.879	0.78	0.89	39	48	6	11
	PubChem-RF	0.750	0.871	0.68	0.81	34	44	10	16
	Graph-LR	0.779	0.870	0.76	0.80	38	43	11	12
	PubChem-Tree	0.827	0.867	0.82	0.83	41	45	9	9
External test set	Ext-RF	0.840	0.930	0.75	0.92	9	12	1	3
	Ext-LR	0.840	0.974	0.75	0.92	9	12	1	3
	Ext-ANN	0.800	0.962	0.67	0.92	8	12	1	4
	Ext-SVM	0.880	0.904	0.83	0.92	10	12	1	2
	Graph-RF	0.880	0.920	0.92	0.85	11	11	2	1
	PubChem-LR	0.840	0.936	0.92	0.77	11	10	3	1
	Ext-Tree	0.880	0.901	0.83	0.92	10	12	1	2
	PubChem-RF	0.800	0.917	0.75	0.85	9	11	2	3
	Graph-LR	0.840	0.936	0.75	0.92	9	12	1	3
	PubChem-Tree	0.640	0.667	0.67	0.62	8	8	5	4

^1^ CA, classification accuracy; AUC, the area under the ROC curve; SE, sensitivity; SP, specificity; TP, the number of true positive compounds; TN, the number of true negative compounds; FP, the number of false positive compounds; FN, the number of true negative compounds.

**Table 3:** Representative privileged substructures obtained from PubChem fingerprint responsible for O^6^-methylguanine-DNA methyltransferase (MGMT) inhibition in base analogs.

No.	Privileged Substructures	General Substructures	Representative Compounds	IG	FP	FN
FP297	C-Br	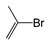	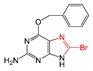	0.096	2.08(11)	0(0)
FP327	C(~Br)(~C) ^1^			0.087	2.08(11)	0(0)
FP328	C(~Br)(~C)(~C)			0.087	2.08(11)	0(0)
FP330	C(~Br)(:C) ^2^			0.087	2.08(11)	0(0)
FP43	≥1 Br			0.096	2.08(11)	0(0)
FP509	Br-C:C-C	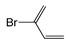	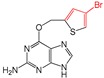	0.078	2.08(9)	0(0)
FP554	Br-C-C-C			0.078	2.08(9)	0(0)
FP670	Br-C:C:C-C			0.078	2.08(9)	0(0)
FP421	C=S	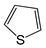	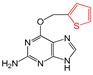	0.078	2.08(9)	0(0)
FP471	S:C:C:C			0.078	2.08(9)	0(0)
FP480	C:S:C-C			0.078	2.08(9)	0(0)
FP513	S:C:C-[#1]			0.078	2.08(9)	0(0)
FP532	S-C:C-[#1]			0.078	2.08(9)	0(0)
FP699	O-C-C-C-C-C(C)-C	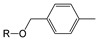	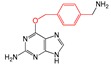	0.096	2.08(11)	0(0)
FP776	CC1CCC(C)CC1			0.064	2.08(11)	0(0)
FP188	≥2 saturated or aromatic heteroatom-containing ring size 6	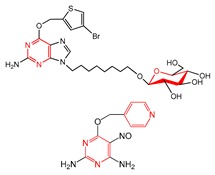		0.081	1.93(13)	0.14(1)
FP648	O=N-C:C-N	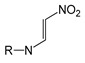	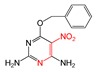	0.073	1.92(12)	0.15(1)
FP260	≥3 hetero-aromatic rings	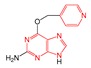		0.056	1.89(10)	0.18(1)
FP713	Cc1ccc(C)cc1	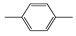	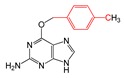	0.056	1.89(10)	0.18(1)
FP697	C-C-C-C-C-C(C)-C	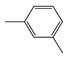	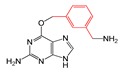	0.048	1.87(9)	0.19(1)

^1^ “~“represent “regardless of bond order”; ^2^ “:” represents bond aromaticity.

## Supplementary Materials

Supplementary materials is available online. Figure S1 is description of correlated descriptors for all compounds in this study. Table S1 is high correlated descriptors. Table S2 is fitting and internal validation parameters of initial QSAR models selected by MCDM. Table S3 is fitting and internal validation parameters of further QSAR models selected by MCDM. Table S4 is statistical description of compounds used in the training and test sets. Table S5 is detailed results of IG values and frequencies of each fragment occurred in the “P” and “N” classes. Table S6 is chemical structures and experimental activity values (pIC50) of base analogs as MGMT inhibitors.
