# Supplementary material for: In Silico Prediction of O6-Methylguanine-DNA Methyltransferase Inhibitory Potency of Base Analogs with QSAR and Machine Learning Methods
Source: Molecules. 2018 Nov 6;23(11):2892. doi: 10.3390/molecules23112892 (PMC6278368; doi:10.3390/molecules23112892)
Supplement: Supplementary file 1 [file molecules-23-02892-s001.zip › molecules-384067 supple revised3.1/Supplementary materials-Figure S1 Table S2 S3 S4 S6.pdf]

Supplementary Materials

In Silico Prediction of O^6-Methylguanine-DNA Methyltransferase Inhibitory Potency of Base analogs with QSAR and Machine Learning Methods

Guohui Sun [1,*], Tengjiao Fan [1], Xiaodong Sun [1], Yuxing Hao [1], Xin Cui [1], Lijiao Zhao [1,*], Ting Ren [1], Yue Zhou [2], Rugang Zhong [1] and Yongzhen Peng [3]

[1] Beijing Key Laboratory of Environmental & Viral Oncology, College of Life Science & Bioengineering, Beijing University of Technology, Beijing 100124, PR China; sunguohui@bjut.edu.cn (G.S.); fantengjiao2014@emails.bjut.edu.cn (T.F.); sunxd@emails.bjut.edu.cn (X.S.); haoyuxing@emails.bjut.edu.cn (Y.H.); cuixin1201@emails.bjut.edu.cn (X.C.); zhaolijiao@bjut.edu.cn (L.J.); renting@bjut.edu.cn (T.R.); lifesci@bjut.edu.cn (R.Z.)

[2] State Key Laboratory of Bioactive Substances and Functions of Natural Medicines, Institute of Materia Medica, Chinese Academy of Medical Sciences & Peking Union Medical College, 2A Nanwei Road, Beijing 100050, PR China; ; zhouyue@imm.ac.cn (Y.Z.)

[3] National Engineering Laboratory for Advanced Municipal Wastewater Treatment & Reuse Technology, Engineering Research Center of Beijing, Beijing University of Technology, Beijing 100124, PR China; pyz@bjut.edu.cn (Y.P.)

Corresponding author: Guohui Sun and Lijiao Zhao

Tel.: +86-10-6739-6180 (G.S.); +86-10-6739-1667 (L.J.)

E-mail address: sunguohui@bjut.edu.cn (G.S.) and zhaolijiao@bjut.edu.cn (L.J.)

Figure S1

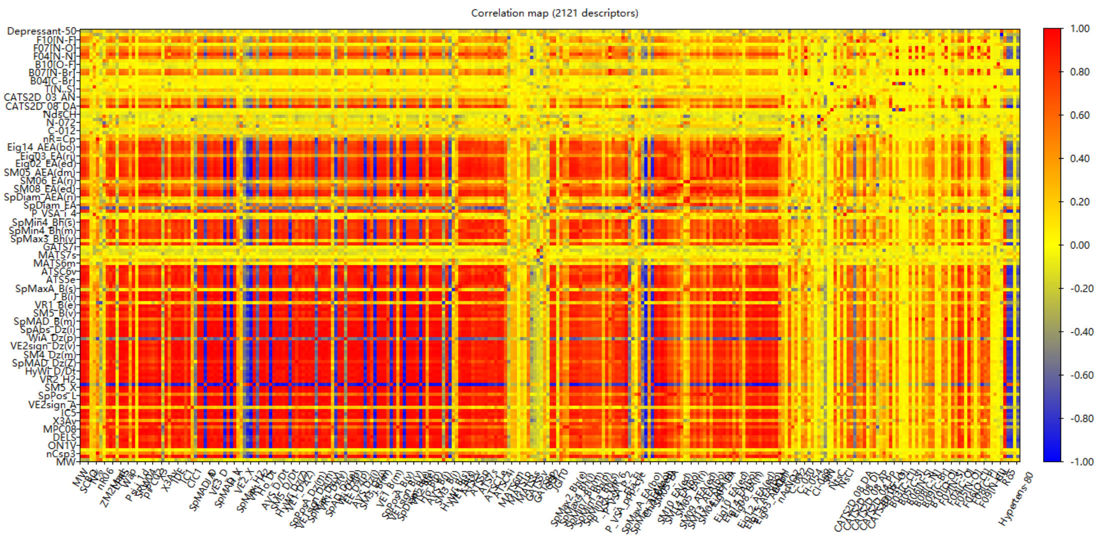

Figure S1. Description of correlated descriptors for all compounds in this study.

Table S2. Fitting and internal validation parameters of initial QSAR models selected by MCDM.

Fitting and internal validation parameters

No.	Model No.	Number of descriptors	Descriptors	R^2	R^2_{adj}	$RMSE_{tr}$	CCC_{tr}	F	Q^2_{loo}	$RMSE_{cv}$	CCC_{cv}	R^2_{Yscr}	Q^2_{Yscr}
**1**	**54**	**5**	**J_Dz(p), VE1sign_B(i), GATS1p, JGI4, CATS2D_05_AA**	**0.68**	**0.66**	**0.87**	**0.81**	**32.9383**	**0.6367**	**0.9338**	**0.7850**	**0.0591**	**-0.0978**
2	56	5	J_Dz(p), VE3sign_B(s), GATS2m, GATS1p, CATS2D_07_PL	0.68	0.66	0.88	0.81	32.60	0.64	0.93	0.79	0.06	-0.10
3	58	5	J_Dz(p), VE3sign_B(s), GATS1p, GATS2p, CATS2D_07_PL	0.67	0.64	0.89	0.80	31.33	0.62	0.94	0.78	0.06	-0.10
4	59	5	J_Dz(p), SpMAD_B(m), JGI4, CATS2D_05_AA, CATS2D_07_PL	0.67	0.65	0.89	0.80	31.28	0.63	0.94	0.78	0.06	-0.10

External validation parameters

No.	Model No.	Number of descriptors	R^2_{ext}	$RMSE_{ext}$	Q^2_{F1}	Q^2_{F2}	Q^2_{F3}	CCC_{ext}	MAE_{ext}	r^2_m	Δr^2_m	k	k′	$(R^2_{ext} - R_0^2)/R^2_{ext}$	$(R^2_{ext} - R'^2_0)/R^2_{ext}$
**1**	**54**	**5**	**0.78**	**0.75**	**0.76**	**0.76**	**0.77**	**0.88**	**0.58**	**0.69**	**0.02**	**0.97**	**1.01**	**0.01**	**0.02**
2	56	5	0.77	0.82	0.71	0.71	0.72	0.87	0.71	0.68	0.19	0.98	0.99	0	0.08
3	58	5	0.75	0.79	0.73	0.73	0.74	0.87	0.66	0.65	0.01	0.99	0.98	0.03	0.02
4	59	5	0.73	0.90	0.65	0.65	0.66	0.85	0.75	0.63	0.10	1.02	0.95	0.01	0.06

Table S3. Fitting and internal validation parameters of further QSAR models selected by MCDM.

Fitting and internal validation parameters

No.	Model No.	Number of descriptors	Descriptors	R^2	R^2_{adj}	$RMSE_{tr}$	CCC_{tr}	F	Q^2_{loo}	$RMSE_{cv}$	CCC_{cv}	R^2_{Yscr}	Q^2_{Yscr}
**1**	**4**	**9**	**VE3sign_X, J_Dz(p), SpPosA_B(p), VE3sign_B(s), MATS1i, JGI4, nArNH2, CATS2D_07_DA, B09[C-C]**	**0.87**	**0.85**	**0.53**	**0.93**	**44.06**	**0.82**	**0.62**	**0.91**	**0.13**	**-0.22**
2	13	9	VE3sign_X, J_Dz(p), SpPosA_B(p), VE3sign_B(s), MATS1i, GATS6e, JGI4, B05[N-O], B09[C-C]	0.87	0.85	0.54	0.93	41.59	0.82	0.62	0.90	0.13	-0.21
3	22	8	VE3sign_X, J_Dz(p), SpPosA_B(p), VE3sign_B(s), MATS1i, JGI4, B09[C-C], F05[N-O]	0.85	0.83	0.57	0.92	43.01	0.81	0.64	0.90	0.12	-0.19
4	23	8	VE3sign_X, J_Dz(p), SpPosA_B(p), MATS1i, GATS1p, JGI4, CATS2D_07_DA, B09[C-C]	0.85	0.83	0.57	0.92	42.89	0.81	0.64	0.90	0.12	-0.18
5	25	8	VE3sign_X, J_Dz(p), SpPosA_B(p), VE3sign_B(s), MATS1i, JGI4, B05[N-O], B09[C-C]	0.85	0.83	0.57	0.92	42.49	0.81	0.65	0.90	0.12	-0.19

External validation parameters

No.	Model No.	Number of descriptors	R^2_{ext}	$RMSE_{ext}$	Q^2_{F1}	Q^2_{F2}	Q^2_{F3}	CCC_{ext}	MAE_{ext}	r^2_m	Δr^2_m	k	k′	$(R^2_{ext} - R_0^2)/R^2_{ext}$	$(R^2_{ext} - R'^2_0)/R^2_{ext}$
**1**	**4**	**9**	**0.69**	**0.79**	**0.67**	**0.67**	**0.71**	**0.83**	**0.70**	**0.58**	**0.01**	**0.99**	**0.99**	**0.04**	**0.03**
2	13	9	0.69	0.77	0.6900	0.69	0.73	0.83	0.71	0.57	0.16	0.98	1.00	0.12	0.004
3	22	8	0.62	0.85	0.62	0.62	0.67	0.78	0.75	0.49	0.17	0.99	0.99	0.19	0.01
4	23	8	0.55	0.97	0.50	0.50	0.57	0.74	0.84	0.41	0.05	0.98	0.98	0.16	0.08
5	25	8	0.59	0.9048	0.57	0.57	0.63	0.77	0.81	0.46	0.10	0.98	0.99	0.16	0.04

Table S4. Statistical description of compounds used in the training and test sets

Data sets	Inhibitors	Non-inhibitors	Total
**Training set**	50	54	104
**External test set**	12	13	25
**Total**	62	67	129

Table S6. Chemical structures and experimental activity values (pIC_{50}) of base analogs as MGMT inhibitors.

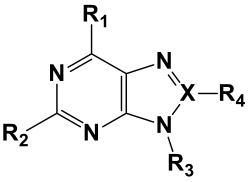

1~3, 5~17, 23~40, 42~117

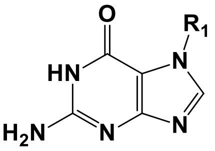

4

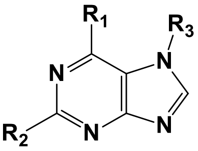

18~22, 41

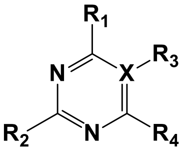

118~134

General molecular structures of all compounds used in this study.

Comp.	R_1	R_2	R_3	R_4	X	pED_{50}
1[a]	$-OCH_3$	$-NH_2$	H	H	C	3.46(N[1])
2	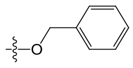	$-NH_2$	H	H	C	6.70(P[2])
3	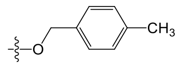	$-NH_2$	H	H	C	6.70(P)
4[b]	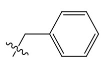	/	/	/	/	3.40(N)
5	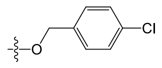	$-NH_2$	H	H	C	6.70(P)
6[b,c]	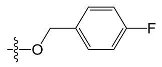	$-NH_2$	H	H	C	6.70(P)
7[a]	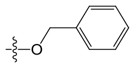	$-NH_2$	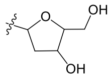	H	C	5.70(P)
8	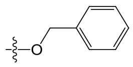	$-NH_2$	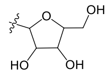	H	C	4.96(N)
9	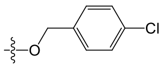	$-NH_2$	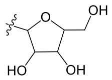	H	C	5.00(P)
10[c]	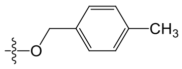	$-NH_2$	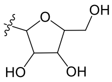	H	C	5.05(P)
11[a,c]	$-OCH_2CH{=}CH_2$	$-NH_2$	H	H	C	4.70(N)
12[c]	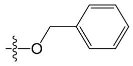	$-NH_2$	$-CH_2COOCH_2CH_3$	H	C	4.52(N)
13	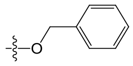	$-NH_2$	$-CH_2COOH$	H	C	3.80(N)
14[b]	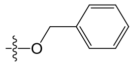	$-NH_2$	$-CH_2C{\equiv}CH$	H	C	4.89(N)
15[a]	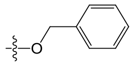	$-NH_2$	$-CH_2CONH_2$	H	C	4.33(N)
16[a]	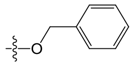	$-NH_2$	$-CH_2CH(OH)CH_2-CH_3$	H	C	4.89(N)
17[a,c]	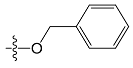	H	H	H	C	4.07(N)
18	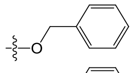	$-NH_2$	$-CH_2COOCH_2CH_3$	/	/	3.40(N)
19	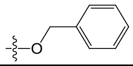	$-NH_2$	$-CH_2COOH$	/	/	3.40(N)

Table S3. *Cont.*

Comp.	R_1	R_2	R_3	R_4	X	pED_{50}
20[c]	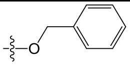	$-NH_2$	$-CH_2C≡CH$	/	/	3.40(N)
21[a]	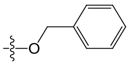	$-NH_2$	$-CH_2CONH_2$	/	/	3.40(N)
22[b]	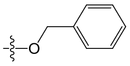	$-NH_2$	$-CH_2CH(OH)CH_2CH_3$	/	/	3.40(N)
23	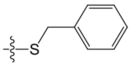	$-NH_2$	H	H	C	3.40(N)
24[c]	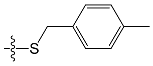	$-NH_2$	H	H	C	3.40(N)
25	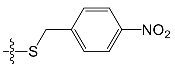	$-NH_2$	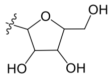	H	C	3.40(N)
26[a]	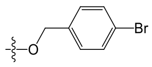	$-NH_2$	H	H	C	6.52(P)
27	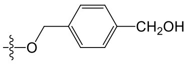	$-NH_2$	H	H	C	6.52(P)
28[a]	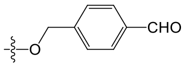	$-NH_2$	H	H	C	6.30(P)
29	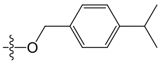	$-NH_2$	H	H	C	6.30(P)
30	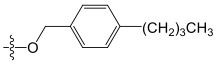	$-NH_2$	H	H	C	5.40(P)
31[c]	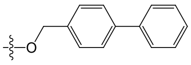	$-NH_2$	H	H	C	6.52(P)
32	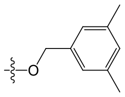	$-NH_2$	H	H	C	6.00(P)
33	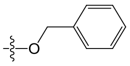	$-NH_2$	$-COCH_3$	H	C	6.40(P)
34	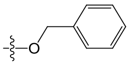	$-NH_2$	$-CH_3$	H	C	5.59(P)
35	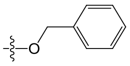	$-NH_2$	$-CH_2OCOC(CH_3)_3$	H	C	5.51(P)
36	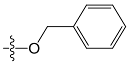	$-NH_2$	$-CH_2CH(OH)CH_2Cl$	H	C	4.74(N)
37	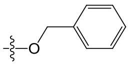	$-NH_2$	$-CH_2CH(OH)CH_2NH-CH(CH_3)_2$	H	C	3.97(N)
38	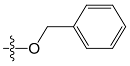	$-NH_2$	$-CH_2CH(OH)CH_2NH-C(CH_3)_3$	H	C	3.97(N)
39[a]	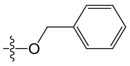	$-NH_2$	$-CH_2CH(OH)CH_2O-CH(CH_3)_2$	H	C	5.15(P)
40[b]	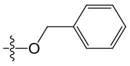	$-NHCOCH_3$	H	H	C	4.62(N)
41[a]	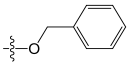	$-NH_2$	$-CH_3$	/	/	4.28(N)
42	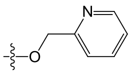	$-NH_2$	H	H	C	4.24(N)
43	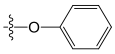	$-NH_2$	H	H	C	3.52(N)
44	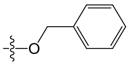	$-NH_2$	H	$-NH_2$	C	6.15(P)
45	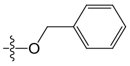	$-NH_2$	H	$-CH_3$	C	6.52(P)
46[b]	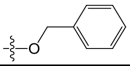	$-NH_2$	H	-OH	C	6.52(P)

Table S3. *Cont.*

Comp.	R_1	R_2	R_3	R_4	X	pED_{50}
47[a,c]	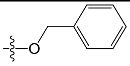	$-NH_2$	H	-Br	C	7.10(P)
48	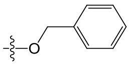	$-NH_2$	H	/	N	7.15(P)
49	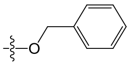	$-NH_2$	H	-F	C	6.40(P)
50[c]	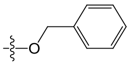	-OH	H	H	C	4.22(N)
51	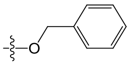	-OH	H	-OH	C	4.60(N)
52[b]	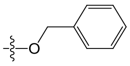	-F	H	H	C	4.32(N)
53[b,c]	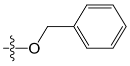	$-NHCOCH_3$	H	-OH	C	4.19(N)
54[a]	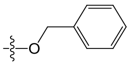	$-NHCH_3$	H	H	C	3.80(N)
55[b]	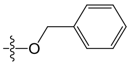	$-N(CH_3)_2$	H	H	C	3.70(N)
56	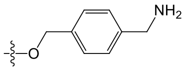	$-NH_2$	H	H	C	6.82(P)
57[b]	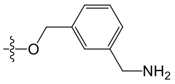	$-NH_2$	H	H	C	6.96(P)
58	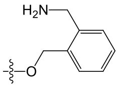	$-NH_2$	H	H	C	4.28(N)
59[b]	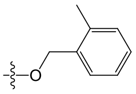	$-NH_2$	H	H	C	3.91(N)
60[a,c]	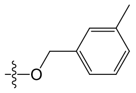	$-NH_2$	H	H	C	6.77(P)
61	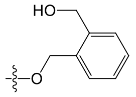	$-NH_2$	H	H	C	3.18(N)
62	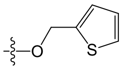	$-NH_2$	H	H	C	8.52(N)
63	$-OCH_2CH_3$	$-NH_2$	H	H	C	<3.00(N)
64	$-O(CH_2)_2CH_3$	$-NH_2$	H	H	C	<3.00(N)
65[a,c]	$-O(CH_2)_3CH_3$	$-NH_2$	H	H	C	3.31(N)
66	$-O(CH_2)_2CH-(CH_3)_2$	$-NH_2$	H	H	C	<3.00(N)
67	$-O(CH_2)_4CH_3$	$-NH_2$	H	H	C	<3.00(N)
68	$-O(CH_2)_5CH_3$	$-NH_2$	H	H	C	3.26(N)
69[c]	$-O(CH_2)_6CH_3$	$-NH_2$	H	H	C	3.30(N)
70	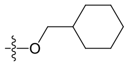	$-NH_2$	H	H	C	<3.00(N)
71	$-OCH_2CH(=CH_2)-CH_3$	$-NH_2$	H	H	C	4.60(N)
72	$-O(CH_2)_4CH=CH_2$	$-NH_2$	H	H	C	/
73	(*Z*)-$O(CH_2)_2CH=CHCH_2CH_3$	$-NH_2$	H	H	C	/
74[a]	(*E*)-$O(CH_2)_2CH=CHCH_2CH_3$	$-NH_2$	H	H	C	<3.00(N)
75	$-O(CH_2)_2C{\equiv}CCH_2-CH_3$	$-NH_2$	H	H	C	/

Table S3. *Cont.*

Comp.	R_1	R_2	R_3	R_4	X	pED_{50}
76	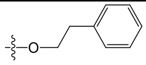	$-NH_2$	H	H	C	3.26(N)
77[c]	$-OCH_2CH(=CH_2)-CH_2CH_3$	$-NH_2$	H	H	C	4.80(N)
78	$-OCH_2CH(=CH_2)-CH_2(CH_3)_2$	$-NH_2$	H	H	C	<3.00(N)
79	$-OCH_2C(CH_3)=$ $CHCH_3$	$-NH_2$	H	H	C	/
80	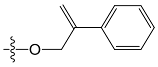	$-NH_2$	H	H	C	4.11(N)
81	$-OCH_2C\equiv CH$	$-NH_2$	H	H	C	4.70(N)
82	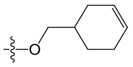	$-NH_2$	H	H	C	<3.00(N)
83	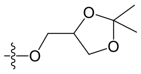	$-NH_2$	H	H	C	<3.00(N)
84	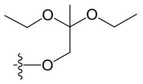	$-NH_2$	H	H	C	<3.00(N)
85[a]	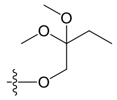	$-NH_2$	H	H	C	<3.00(N)
86	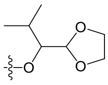	$-NH_2$	H	H	C	/
87	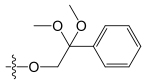	$-NH_2$	H	H	C	<3.00(N)
88[b]	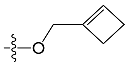	$-NH_2$	H	H	C	6.26(P)
89	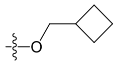	$-NH_2$	H	H	C	<3.00(N)
90	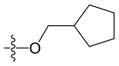	$-NH_2$	H	H	C	<3.00(N)
91[b,c]	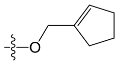	$-NH_2$	H	H	C	6.41(P)
92	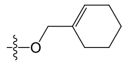	$-NH_2$	H	H	C	5.80(P)
93[b]	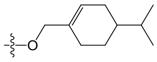	$-NH_2$	H	H	C	5.59(P)
94	$-OCH_2COCH_3$	$-NH_2$	H	H	C	3.72(N)
95	$-OCH_2COCH_2-$ CH_3	$-NH_2$	H	H	C	3.82(N)
96	$-OCH_2COCH-$ $(CH_3)_2$	$-NH_2$	H	H	C	<3.00(N)
97[a,c]	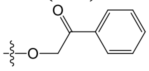	$-NH_2$	H	H	C	<3.00(N)
98[b]	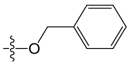	$-NH_2$	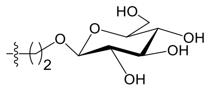	H	C	5.10(P)
99[a]	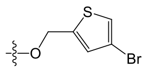	$-NH_2$	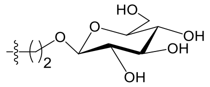	H	C	6.66(P)
100	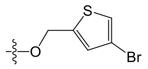	$-NH_2$	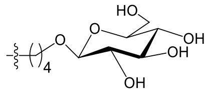	H	C	6.82(P)
101	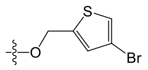	$-NH_2$	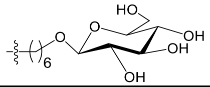	H	C	6.82(P)

Table S3. *Cont.*

Comp.	R_1	R_2	R_3	R_4	X	pED_{50}
102	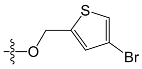	$-NH_2$	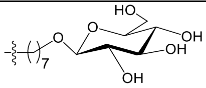	H	C	7.10(P)
102	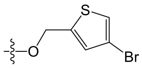	$-NH_2$	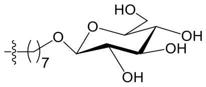	H	C	7.10(P)
103	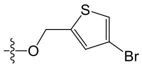	$-NH_2$	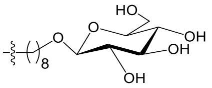	H	C	8.00(P)
104[b,c]	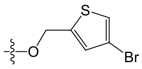	$-NH_2$	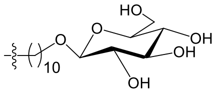	H	C	8.00(P)
105[a]	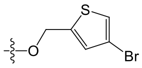	$-NH_2$	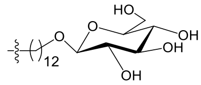	H	C	8.00(P)
106	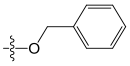	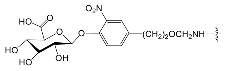	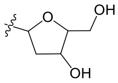	H	C	4.30(N)
107[c]	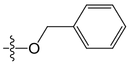	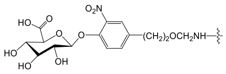	H	H	C	4.30(N)
108	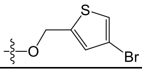	$-NH_2$	H	H	C	8.54(P)
**ED_{50} values of 109~134 were determined on HeLa S3 cell extracts**						
109	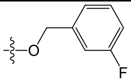	$-NH_2$	H	H	C	P
110	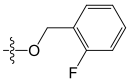	$-NH_2$	H	H	C	P
111	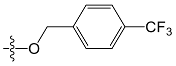	$-NH_2$	H	H	C	N
112	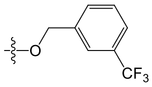	$-NH_2$	H	H	C	P
113	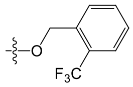	$-NH_2$	H	H	C	P
114	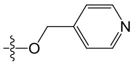	$-NH_2$	H	H	C	N
115[c]	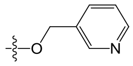	$-NH_2$	H	H	C	P
116[c]	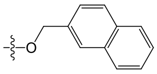	$-NH_2$	H	H	C	P
117[c]	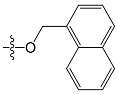	$-NH_2$	H	H	C	N
118	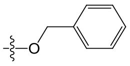	H	$-NO_2$	$-NH_2$	C	N
119	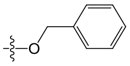	$-NH_2$	H	$-NH_2$	C	N
120	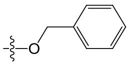	$-NH_2$	$-NH_2$	$-NH_2$	C	P
121[c]	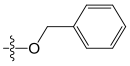	$-NH_2$	-NO	$-NH_2$	C	P
122	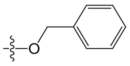	$-NH_2$	$-NO_2$	$-NH_2$	C	P
123[c]	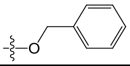	$-NH_2$	-Br	$-NH_2$	C	P

Table S3. *Cont.*

Comp.	R_1	R_2	R_3	R_4	X	pED_{50}
124	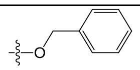	$-NH_2$	$-NO_2$	H	C	P
125	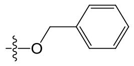	$-NH_2$	$-NO_2$	$-CH_3$	C	P
126[c]	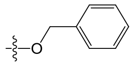	$-NH_2$	/	$-NH_2$	N	P
127	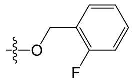	$-NH_2$	$-NO_2$	$-NH_2$	C	P
128	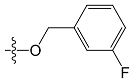	$-NH_2$	$-NO_2$	$-NH_2$	C	P
129	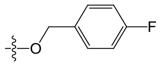	$-NH_2$	$-NO_2$	$-NH_2$	C	P
130	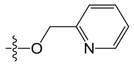	$-NH_2$	$-NO_2$	$-NH_2$	C	P
131	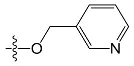	$-NH_2$	$-NO_2$	$-NH_2$	C	P
132	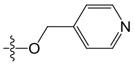	$-NH_2$	$-NO_2$	$-NH_2$	C	P
133	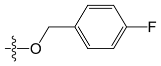	$-NH_2$	-NO	$-NH_2$	C	P
134	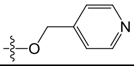	$-NH_2$	-NO	$-NH_2$	C	P

[1] "N" represents MGMT non-inhibitors;

[2] "P" represents MGMT inhibitors;

[a] Test set in initial QSAR study (Model I);

[b] Test set in further QSAR study (Model II);

[c] Test set in classification study.
